# Sex-specific stress response and HMGB1 release in pulmonary endothelial cells

**DOI:** 10.1371/journal.pone.0231267

**Published:** 2020-04-09

**Authors:** Marina Zemskova, Sergey Kurdyukov, Joel James, Nolan McClain, Ruslan Rafikov, Olga Rafikova

**Affiliations:** Division of Endocrinology, Department of Medicine, University of Arizona, Tucson, AZ, United States of America; Stanford University, UNITED STATES

## Abstract

Women are known to be associated with a higher susceptibility to pulmonary arterial hypertension (PAH). In contrast, male PAH patients have a worse survival prognosis. In this study, we investigated whether the contribution of sex goes beyond the effects of sex hormones by comparing the ability of isolated male and female pulmonary endothelial cells to respire, proliferate and tolerate the stress. Mouse lung endothelial cells (MLEC) were isolated from the lungs of male and female 3-week old mice. Male MLEC showed an increased basal mitochondrial respiration rate, elevated maximal respiration, a significantly greater level of mitochondrial polarization, and a higher rate of proliferation. Exposure of cells to hypoxia (2% of O_2_ for 24 hours) induced a strong apoptotic response in female but not male MLEC. In contrast, treatment with mitochondrial respiratory Complex III inhibitor Antimycin A (AA, 50μM) mediated severe necrosis specifically in male MLEC, while female cells again responded primarily by apoptosis. The same effect with female cells responding to the stress by apoptosis and male cells responding by necrosis was confirmed in starved pulmonary endothelial cells isolated from human donors. Elevated necrosis seen in male cells was associated with a significant release of damage-associated alarmin, HMGB1. No stimuli induced a significant elevation of HMGB1 secretion in females. We conclude that male cells appear to be protected against mild stress conditions, such as hypoxia, possibly due to increased mitochondrial respiration. In contrast, they are more sensitive to impaired mitochondrial function, to which they respond by necrotic death. Necrosis in male vascular cells releases a significant amount of HMGB1 that could contribute to the pro-inflammatory phenotype known to be associated with the male gender.

## 1. Introduction

Sex is a known contributing factor to many cardiovascular diseases. Due to the protective effects of female sex hormones females are generally found to be less affected or have a better prognosis. However, in pulmonary arterial hypertension (PAH), the female gender is known to be associated with a higher susceptibility to the disease. This effect, known as the “estrogen paradox,” is under a close investigation by many research groups [1–4].

Although males have a significantly lower prevalence in PAH, they show a conspicuously worse survival prognosis [5, 6]. The poor survival of male PAH patients is commonly attributed to the progressive development of right ventricle (RV) failure [6], possibly due to the absence of cardioprotection mediated by female hormones. Besides, it has been recently discovered that the male gender is associated with a considerably more severe inflammatory profile compared to females [7]. Thus, many inflammatory conditions, including acute respiratory distress syndrome (ARDS) [8], meningitis [9], and myocarditis [10] were reported to have more severe forms and poorer prognosis in males. Males commonly have a worse recovery from surgical procedures [11], show higher production of inflammatory markers [12], and a higher rate of infection-induced mortality in general [7]. Since inflammation is an established contributor to PAH pathogenesis, the increased inflammatory reactions seen in males could significantly contribute to the PAH progression and outcome.

Indeed, the inflammation-associated forms of PAH, such as HIV-associated PAH, have an inverted male to female ratio 7.7:1 [13]. Our previously published studies that used Sugen/Hypoxia-induced rat model of PAH showed that male rats have worse survival, more severe intravascular and perivascular inflammation, and activation of inflammatory pathways in lungs [14, 15]. None of these changes were evident in PAH female rats. The less pronounced inflammatory changes in PAH females were recently associated with the increased functionality of regulatory T cell (Treg) [16]. The ability of T regs to suppress over-activation of the immune system, inflammation, and perivascular infiltration of pulmonary vessels with inflammatory cells protects females against PAH.

Although sex-mediated effects are classically viewed through the prism of sex hormones, the last research [16] and a few other recent reports [17] suggest the presence of non-hormonal mechanisms that are responsible for manifestation of the sex difference. Therefore, in this study, we were interested in investigating whether pulmonary endothelial cells omitted from the effects of sex hormones or interactions with other cell types will still preserve the sex difference. Indeed, we confirmed that sex determines the phenotypical difference, even in the isolated cells. In addition to the earlier discussed reports, this discovery suggests the importance of genetic factors in the manifestation of these sex-specific phenotypes. Although identification or validation of such factors was not the focus of this study, we believe that these mechanisms require considerably more attention than they were given before.

## 2. Methods

### 2.1. Isolation and validation of mouse lung endothelial cells

Isolation of MLEC from the murine lung was performed according to the protocol described previously [18] with some modifications. All experimental procedures were approved by the University of Arizona’s Institutional Animal Care and Use Committee. Briefly, 21-day-old male and female mice C57BL/6 randomized by the provider (Charles River, Wilmington, MA) were euthanized using cervical dislocation. After the chest was opened, the left atrium was dissected, and the lungs were perfused with cold sterile 0.9% saline via the right ventricle. Heart and lung block was removed; lungs were separated, minced, and digested in 1mg/ml collagenase (Worthington Biochemical, Lakewood, NJ) for 45min at 37C. Magnetic beads (ThermoFisher, Waltham, MA) labeled with PECAM-1-antibody (BD Pharmingen, San Diego, CA) were used to isolate the MLEC from the collagenase-digested lung cell suspension. After being filtered and washed, the lung cells were plated in 6-well plate and cultured in Dulbecco's modified Eagle's medium (DMEM, GIBCO, ThermoFisher, Waltham, MA) containing 10% FBS (GenClone, Genessee Scientific), 100ug/ml Heparin (Sigma H-3933), 100ug/ml ECGS (Sigma #E2759-15mg, Lot SLBT2205), non-essential amino acids (Sigma, M7145-100ml, Lot RNBF8326), 2mM L-glutamine, sodium pyruvate, and 10mM HEPES and counted as passage P0. After 48–72 h in culture, the lung cells were re-plated in 10cm dishes as passage P1 using the same culture media. At P2, MLEC were collected, resuspended to a concentration of 0.1-1x10^6^ cells in 100ul of PBS, and treated with anti-ICAM2-Alexa Fluor antibody (Invitrogen, 53-1021-82) in dilution 1:100 in the dark. Rat IgG2 iso-control antibodies (eBioscience, 53-4321-80) were used as control. MLEC labeled with anti-ICAM-2 and control antibody were washed, resuspended in PBS, and the purity of cells was analyzed using NovoCyte Flow Cytometer (ACEA Biosciences, San Diego, CA). Four separate isolations of MLEC (N = 5–7 per gender per isolation) were performed from mice obtained from different litters.

### 2.2. Analysis of cell size and proliferation rate

Male and female MLEC at P2 were trypsinized, trypsin was neutralized by adding 10 volumes of media, and cells spun at 300g. Next, cells were resuspended in PBS buffer with 1mM EDTA. Analysis of the cell size was performed using NovoCyte Flow Cytometer (ACEA Biosciences, San Diego, CA). Cell size was measured using forward scatter. The experiment was repeated after each cell isolation. Nuclei of the confluent male and female MLEC were also stained with DAPI using a standard protocol. Microscopy was performed using Leica AM6000 inverted microscope (Leica Microsystems, Wetzlar, Germany) at 40X magnification, as previously described [19].

The cell growth rate was determined using the iCELLigence System (ACEA Biosciences, San Diego, CA). The method is based on measuring real-time changes in electrical impedance that was recorded as the Cell index (CI). The assays were performed according to the manufacturer's instructions, and the CI and the normalized CI were defined as described previously [20]. Briefly, the plates were filled with 200 μl of media and connected to the iCELLigence System to produce a time zero control for each plate and well. After the cells were seeded on the plates and recovered for 30 min, the plates were returned to the iCELLigence system, and the CI was measured every 30 minutes by the installed software for the duration of the experiments.

### 2.3. Mitochondrial polarization

Mitochondrial Membrane Potential Probe JC-1 (TermoFisher Scientific, Waltham, MA), at concentration 2ug/ml, was added to cells and incubated at 37°C for 90 minutes. Cells were collected by trypsinization; trypsin was neutralized with 10 volumes of media, cells were centrifuged at 300g, resuspended in PBS, and analyzed using the NovoCyte Flow Cytometer (ACEA Biosciences). The cells were gated, excluding debris. Standard compensation (the threshold for PE channel—red fluorescent signal corresponding to mitochondria polarization) was established using control cells with mitochondria depolarized by CCCP (carbonyl cyanide 3-chlorophenylhydrazone), a mitochondrial oxidative phosphorylation uncoupler. For this purpose, cells stained with JC-1 were treated with 50μM CCCP (Sigma, St. Louis, MO) for five minutes before cell collection. For fluorescence microscopy, cells were plated in 8-well chambers with coverglass bottom (ThermoFisher) and grown until confluent. JC-1 was added to a final concentration of 2ug/ml. NucBlue Live Ready Probes Reagent (ThermoFisher, Waltham, MA) was used to stain cell nuclei. The cells were incubated in a 37°C humidified incubator with 5% CO_2_ for 90 minutes and analyzed directly in the culture medium since phenol red does not interfere with the fluorescent staining. Microscopy was performed using a Leica AM6000 inverted microscope (Leica Microsystems, Wetzlar, Germany) at 40X magnification using the following filters: rhodamine (excitation/emission = 540/570nm) to detect high mitochondrial membrane potential, FITC (excitation/emission = 485/535nm) to detect low mitochondrial membrane potential, and filter cube DAPI (excitation/emission = 360/460nm) to visualize cell nuclei.

### 2.4. Analysis of mitochondrial respiration

An XF24 Analyzer (Seahorse Biosciences, North Billerica, MA) was used to measure mitochondrial function in intact cells in real-time as previously described[21, 22]. Briefly, male and female MLEC and HPAEC cells were plated in 24-well Seahorse XF24 cell culture Microplates at the seeding density 7.5x10^4^ and 5x10^4^ per well for MLEC and HPAEC correspondingly and allowed to adhere and grow for 16 h in a 37°C humidified incubator with 5% CO_2_. The next day, the culture medium was changed to DMEM, pH 7.4 supplemented with 10mM glucose, 1mM pyruvate, and 2mM glutamine. The culture microplates were incubated in a CO2-free XF prep station at 37°C for 30 min to allow temperature and pH calibration. Cells were washed twice with equilibration media supplied by the manufacturer and left for incubation at 37°C for 30 minutes. The XF Cell Mito Stress Test Kit (#101706–100; Seahorse Biosciences) was used to measure oxygen consumption rate (OCR) at the basal conditions and upon stimulation and expressed in pmoles/min of oxygen consumed. The mitochondrial respiratory parameters were calculated from the OCR values.

### 2.5. Apoptosis and necrosis induction and quantification

Male and female MLEC at passage 2–3 were plated at 70–80% of confluence into 12-well plates and grown overnight in the growth media described above. The next day, the media was changed to the new containing 2% FBS, and the plates were either placed in hypoxia (2% O2) for 24 hours (hypoxic cells), remained in normoxic conditions (untreated cells) or treated with 50μM Antimycin A (AA) for 24 hours (AA treated cells). The level of apoptosis and necrosis in each cell group was measured using Apoptosis and Necrosis Quantification Kit (Biotium Inc., Fremont, CA) according to the kit protocol. Briefly, cells were collected, washed with PBS, resuspended in 50μl of reaction mix containing a buffer, FITC-Annexin V and Ethidium homodimer. After 25 minutes of incubation in the dark at room temperature, samples were diluted with 200μl of the kit 1X buffer. Quantification of apoptotic and necrotic cells was performed using the NovoCyte Flow Cytometer (ACEA Biosciences, San Diego, CA), as previously described [19]. Briefly, early apoptosis was defined as Annexin V^+^/ethdium homodimer-1 (EthD-1)^-^ cells; late apoptosis—as Annexin V^+^/EthD-1^+^; necrotic–as Annexin V^-^/EthD-1^+^ cells. Gating was done based on untreated cells. The same analysis was performed using human pulmonary artery endothelial cells (HPAEC, Lonza, Walkersville, MD) collected from male and female donors (34-year-old Caucasian female, Lot #657513, and 48-year-old Caucasian male, Lot #662151) treated by serum-free VascuLife® EnGS Endothelial Cell Culture Medium (LifeLine, Frederick, MD).

### 2.6. Stress mediated HMGB1 release

High mobility group protein B1 (HMGB1) is a nuclear factor released from dying cells, and, by activating the pattern recognition receptors (PRR), acts as an “alarmin” that initiates inflammation in response to tissue damage. Media collected from MLEC and HPAEC cells, stressed as described above, was used to detect the amount of HMGB1 released using Western Blot analysis (anti-HMGB1 antibody, ab18256 (Abcam, Burlingame, CA) were used in dilution 1:1000, as published)[15]. Briefly, media samples were incubated with 6X Laemmli sample buffer (Boston Bioproducts, Ashland, MA), for 5 min at 95°C, loaded on the 4–20% Mini-PROTEAN TGX Stain-Free gels (Bio-Rad Laboratories, Hercules, CA), and electrophoretically separated and transferred using PowerPac Universal power supply and Trans-Blot Turbo transferring system (Bio-Rad Laboratories). The signal was recorded with the ChemiDoc MP Imaging System (Bio-Rad Laboratories, Hercules, CA) using a chemiluminescent protocol and analyzed using Image Laboratory software. The protein loading was normalized per total sample protein using free stain gels, as previously described [23].

### 2.7. Statistical analysis

Statistical calculations were performed using the GraphPad Prism software version 7.04. The mean value (±SEM) was calculated for all samples, and significance was determined by unpaired t-test or analysis of variance (ANOVA). For ANOVA, Turkey’s multiple comparisons test to compare the selected pairs of columns was used. A value of P < 0.05 was considered significant. The Grubbs test (extreme studentized deviate) was used to determine the significant outliers. This criterion was predetermined before the initiation of the data analysis.

## 3. Results

### 3.1. Isolated MLEC show the sex difference in the size and growth rate

**Fig 1:**
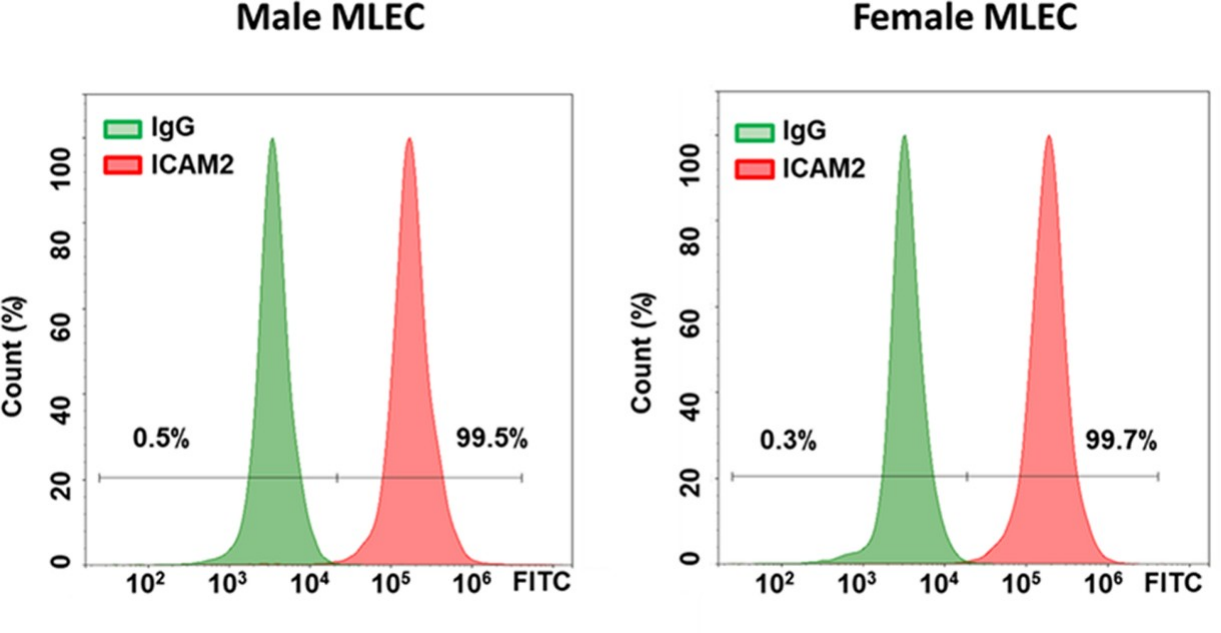
Analysis of isolated male and female MLEC. The percentage of ICAM2 positive male and female MLEC were analyzed. Both sexes showed a comparable and highly homogenous population of MLEC. Representative data out of four independent cell isolations (N = 5–7 per gender per isolation) from mice obtained from different litters.

**Fig 2:**
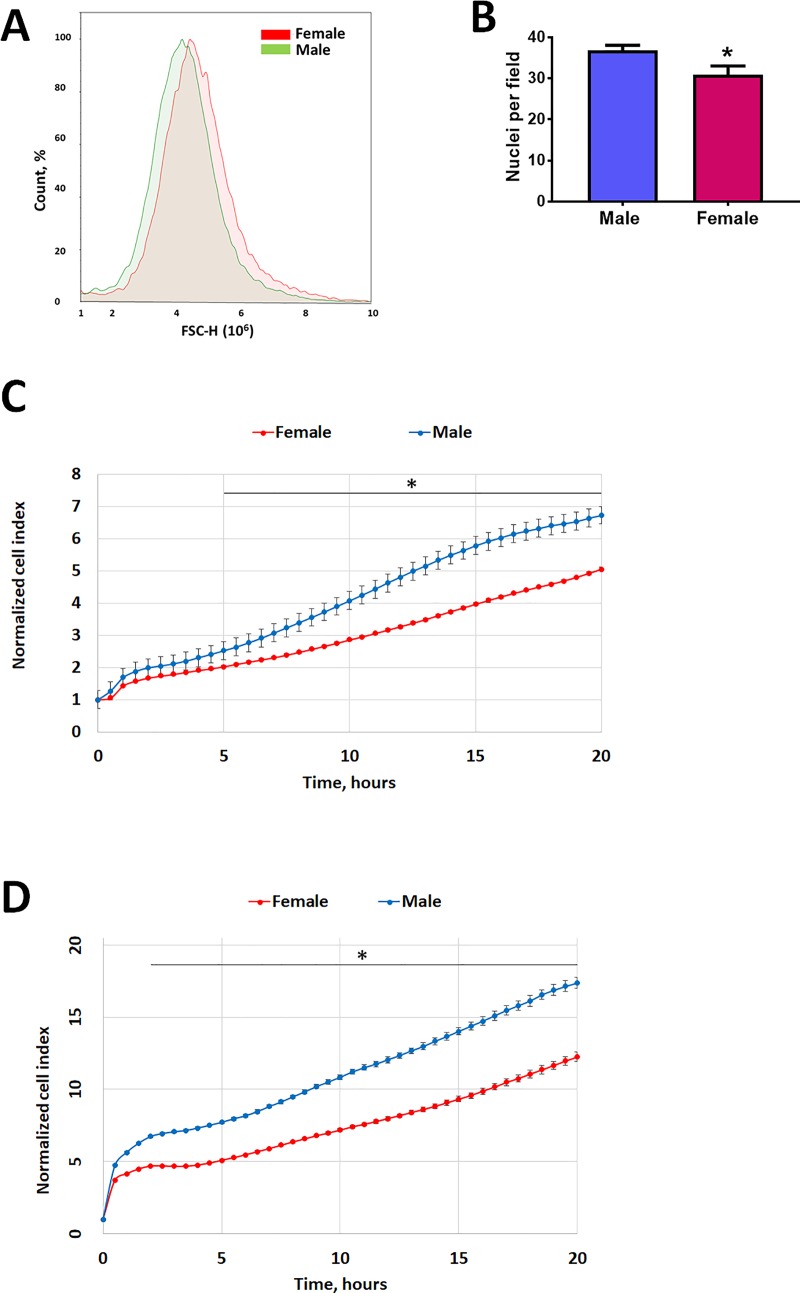
Sex difference in the endothelial size and proliferation rate. MLEC isolated from male mice were found to be smaller (**A, B**) and show a significantly higher proliferation rate (**C**) compared to female MLEC. **A**–representative forward scatter representing MLEC size, N = 5–7. **B**–number of nuclei per field of confluent MLEC at magnification 40X. The higher number of nuclei per field in male MLEC corresponds to the smaller size, N = 8 random fields per sex. Proliferation rate of MLEC (**C**) and HPAEC (**D**) measured using the iCELLigence System. For MLEC, values are means ± SEM; N = 6 repetitions from 5–7 mice isolated for each sex. For HPAEC, one out of two independent experiments is shown, N = 4 repetitions for each sex. *p < 0.05 versus female cells. Statistical analysis was performed using the unpaired t-test.

The purity of MLEC cells was comparable between cells isolated from male and female mice (Fig 1). Nevertheless, these endothelial cells that were no longer exposed to sex hormones or any other stimulation produced by either humoral factors or factors secreted locally from other cell types (like smooth muscle cells, fibroblasts, or inflammatory/immune cells) still preserve a sex-specific phenotype. In particular, we found that male MLEC were smaller in size (Fig 2A and 2B), but had a higher proliferation rate (Fig 2C). Importantly, HPAEC isolated from male and female human donors also showed a significant difference in proliferation rate, with male cells possessing a significantly higher proliferative capacity compared to female (Fig 2D).

### 3.2. Preserved sex difference in mitochondrial function

**Fig 3:**
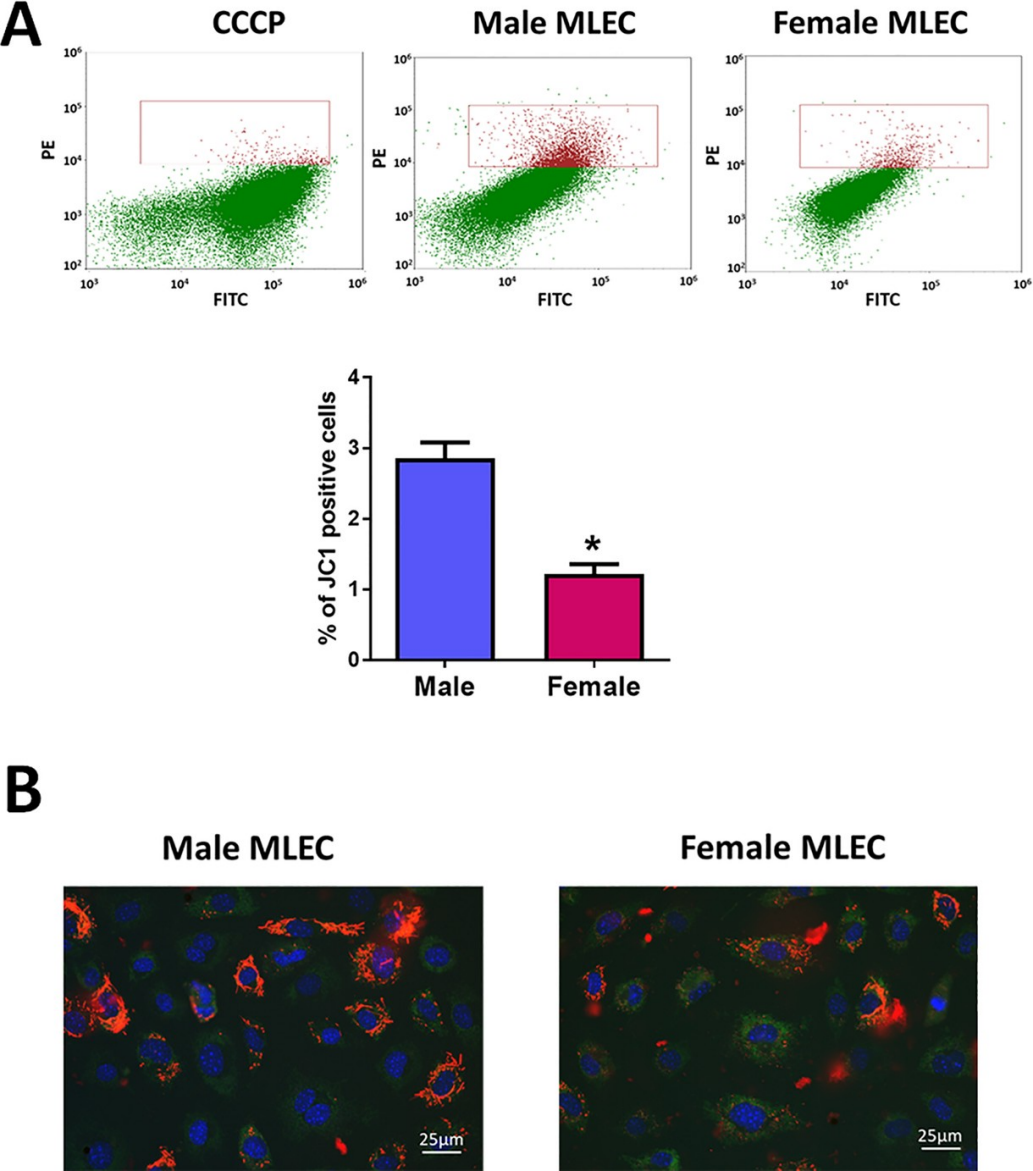
Mitochondrial membrane potential in male and female MLEC. MLEC isolated from male mice show a significantly higher level of mitochondrial polarization. **A**–representative flow cytometry plots and gating strategy (upper panel). MLEC stained by JC1 in the presence of mitochondria depolarizer CCCP were gated to exclude polarized cells. The same gates were used to determine the level of polarization in untreated male and female MLEC. Quantitate analysis of JC1 positive cells (lower panel). Values are means ± SEM; N = 4 repetitions from 5–7 mice isolated for each sex. *p < 0.05 versus male MLEC. Statistical analysis was performed using the unpaired t-test. **B**–Representative fluorescent images show a higher percentage of cells with polarized mitochondrial in male MLEC population.

**Fig 4:**
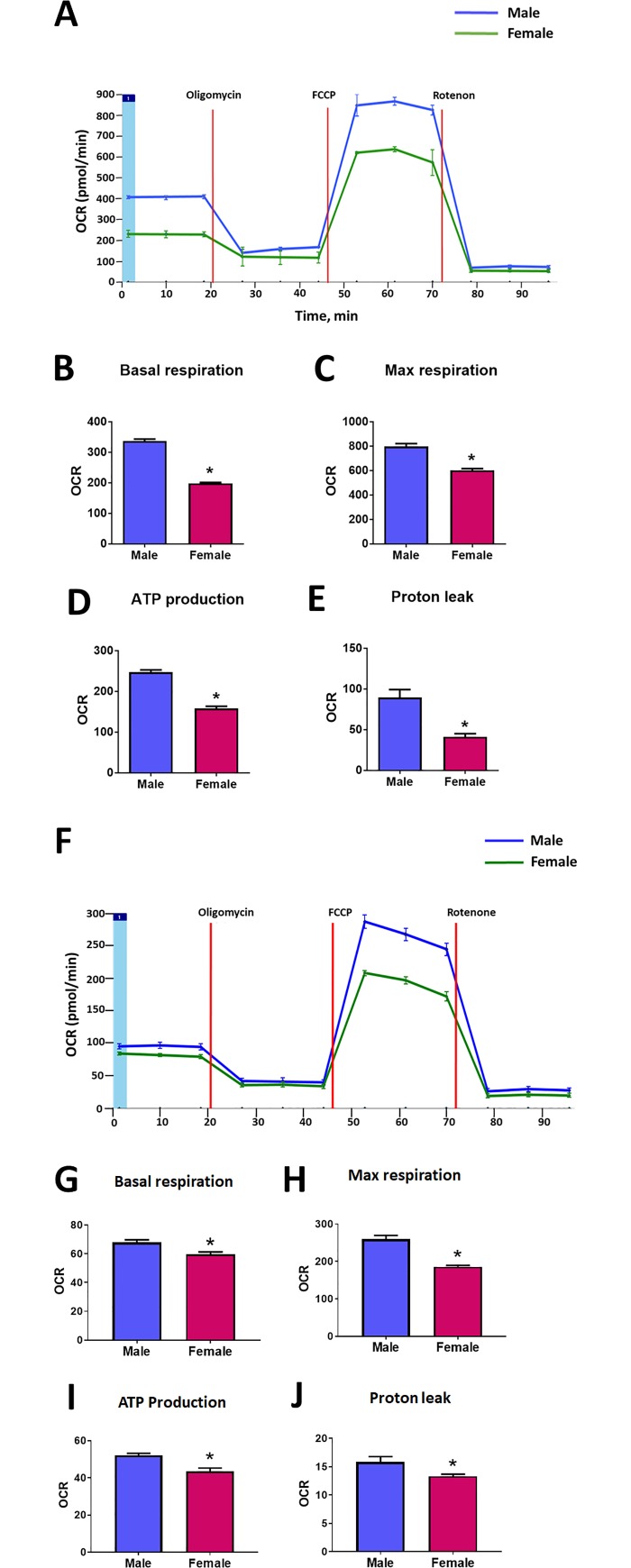
Mitochondrial respiration in MLEC isolated from male and female mice. Mitochondrial function in male and female MLEC and HPAEC was measured using XF24 Analyzer (Seahorse Biosciences, North Billerica, MA). Oligomycin (1μM), FCCP (1μM), and Rotenone and Antimycin A (1μM each) were added at the indicated time points (**A, F**). Basal mitochondrial respiration for MLEC (**B**) and HPAEC (**G**), maximal respiratory capacity for MLEC (**C**) and HPAEC (**H**), ATP production for MLEC (**D**) and HPAEC (**I**), and Proton leak for MLEC (**E**) and HPAEC (**J**) were found to be significantly lower in female MLEC and HPAEC compared to male. Values are means ± SEM. For MLEC, one out of two independent experiments is shown. N = 3 repetitions from 5–7 mice isolated for each sex. For HPAEC, one out of two independent experiments is shown. N = 10 for male and N = 9 repetitions for female donor. *p < 0.05 < 0.05 versus male MLEC. Statistical analysis was performed using the unpaired t-test.

Male and female MLEC showed a significant difference in the number of polarized mitochondria (Fig 3A and 3B). Thus, the number of cells with high mitochondrial potential, quantified by flow cytometry, was found to be about 2 times higher in male MLEC compared to female (Fig 3A). The fluorescent images of MLEC stained with JC1 additionally confirmed that cells with significantly or partially polarized mitochondria appear more often in MLEC isolated from male mice (Fig 3B). This data has strongly correlated with the results obtained from the analysis of mitochondrial respiration (Fig 4). Indeed, assessment of the mitochondrial function using the Seahorse method revealed that there is a significant difference in the level of basal mitochondrial respiration with male MLEC showing ~ 1.7 times higher basal mitochondrial respiration compared to female (Fig 4A and 4B). Taken together, our results suggest that male MLEC have more functional mitochondria compared to females. By performing the mitochondrial stress test, we also discovered that not only basal conditions but also the maximal rate of respiration was significantly different between male and female MLEC (Fig 4A, Fig 4C). The increased functionality of mitochondria in male MLEC corresponded with significantly higher ATP production compared to female MLEC (Fig 4D). Finally, the amount of proton leak as a measure of uncoupling between mitochondrial respiration and ATP production, which could potentially lead to the increased risk of superoxide production, was also significantly elevated in male MLEC (Fig 4E). The same sex-mediated difference in mitochondrial function was observed in the experiment with HPAEC isolated from male and female human donors (Fig 4F–4J). The basal mitochondrial respiration, as well as maximal respiration rate, ATP production, and proton leak were all found to be significantly higher in HPAEC isolated from male donor. Although we noticed that the extent of the sex difference in mitochondrial respiration of mice and human cells was slightly different, we cannot conclude whether this variability is due to the difference in the species, or it is driven by other factors. Such factors could include the difference in the lifestyle among the human donors, their health status, or could be related to the difference in the cell passage.

### 3.3. Sex difference in cellular response to stresses

**Fig 5:**
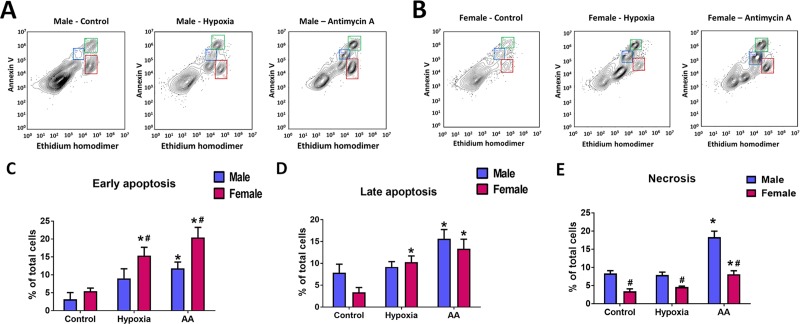
Distinct sex-specific profile of apoptotic and necrotic cell death in MLEC in response to stresses induced by hypoxia or mitochondrial dysfunction. The level of apoptosis and necrosis in male and female MLEC were measured in cells stained by Annexin V and Ethidium homodimer (EthD-1). **A, B**–representative plots showing populations of Annexin V and EthD-1 positive cells in Control MLEC and cells exposed to either hypoxia (24 h of 2% O_2_), or Antimycin A (AA, 50 μM for 24h). **C-E–**quantitative analysis of early apoptosis, late apoptosis, and necrosis in male and female MLEC. Values are means ± SEM. N = 4 repetitions from 5–7 mice isolated for each sex. *p < 0.05 versus same-sex Control cells; **#**p < 0.05 versus male MLEC. Statistical analysis was performed using the unpaired t-test.

We exposed MLEC cells to the two types of stresses relevant to the pathobiology of PAH and other vascular diseases–hypoxia and mitochondrial dysfunction. The representative plots showing populations of dying cells in male and female MLEC in response to these treatment are shown in Fig 5A and 5B. Hypoxia could be seen as relatively mild stress, especially for pulmonary artery endothelial cells that are normally exposed to the reduced level of oxygen in the venous blood. However, we found that exposure of MLEC to 2% of O_2_ for 24 hours was sufficient to induce a significant early and late apoptotic cell death in MLEC isolated from female mice (Fig 5C and 5D). In contrast, no significant difference in the number of apoptotic cells was found in MLEC isolated from males, suggesting a significant sex difference in the cellular response to hypoxia. Hypoxia treatment did not activate necrotic cell death in cells of either sex (Fig 5E). Next, we treated MLEC with Antimycin A (AA), the specific inhibitor of complex III of mitochondrial electron transport chain (ETC), a compound known to induce severe cell stress and cell death [24, 25]. Moreover, prolonged treatment with AA was shown to induce chronic pulmonary vasoconstriction and contribute to PH [22]. Interestingly, the level of early and late apoptosis in MLEC isolated from females was again significantly elevated, although it remained the same as apoptosis occurred in response to hypoxia (Fig 5A, 5B, 5C and 5D). AA treatment induced apoptosis in male cells as well; however, early apoptosis (or amount of Annexin V positive, EthD-1 negative cells) was significantly lower in male cells compared to females (Fig 5C). In contrast, the level of necrosis was significantly increased specifically in males, and only slightly changed in females (Fig 5A, 5B and 5E). Together with the mitochondrial functional data, these results suggest that male cells require the higher activity of mitochondria and are more sensitive to mitochondrial dysfunction compared to female cells. Indeed, cell stress that mediated the inhibition of mitochondrial respiration forced the male cells to die primarily by necrosis, while female cells responded mainly by apoptosis, regardless of the stress type or intensity.

**Fig 6:**
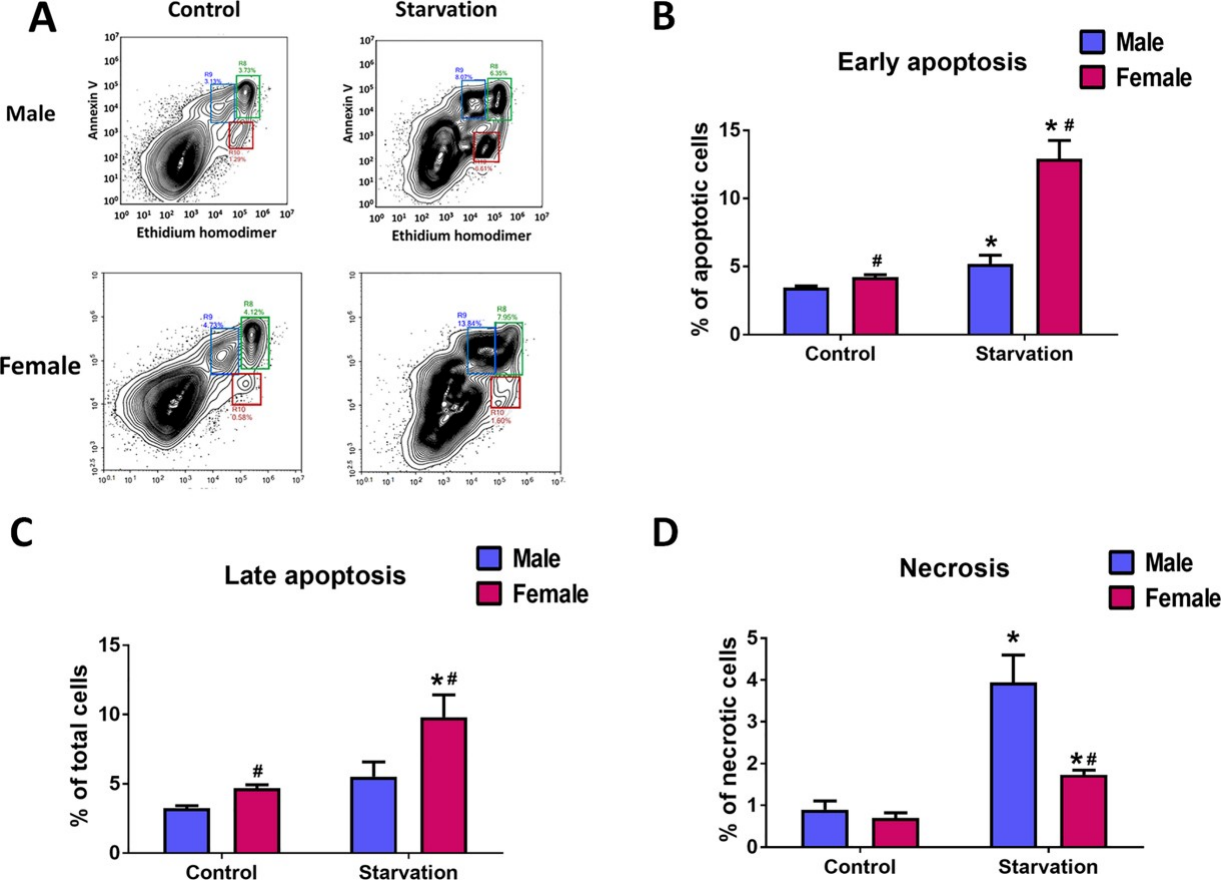
Sex-specific profile of apoptotic and necrotic cell death in HPAEC. Stress in human endothelial cells obtained from age and race matched male and female donors was induced by culturing HPAEC in serum-free media for 48h. **A—**representative plots showing populations of Annexin V and EthD-1 positive cells in Control and starved male and female HPAEC. **B-D–**quantitative analysis of early apoptosis, late apoptosis, and necrosis in male and female HPAEC. Values are means ± SEM. N = 6 for each sex. *p < 0.05 versus same-sex Control cells; **#**p < 0.05 versus male HPAEC. Statistical analysis was performed using unpaired t-test.

To confirm that the significant difference in the type of cell death in response to stress described for males and females is not the unique property of mice endothelial cells, we used human pulmonary artery endothelial cells (HPAEC) isolated from age and race matched male and female donors. We found that starvation of HPAEC by growing them in the serum-free media for 48 hours produces stress severe enough to mediate significant cell death. Importantly, the profile of this cell death was similar to the one seen in mice. Thus, in response to starvation, female HPAEC showed a higher rate of early and late apoptosis (Fig 6A, 6B and 6C), while males showed a higher amount of necrosis (Fig 6A and 6D). We conclude that the discovered sex difference of isolated endothelial cells to tolerate the stress is common for mice and humans.

### 3.4. Stress-induced release of HMGB1

**Fig 7:**
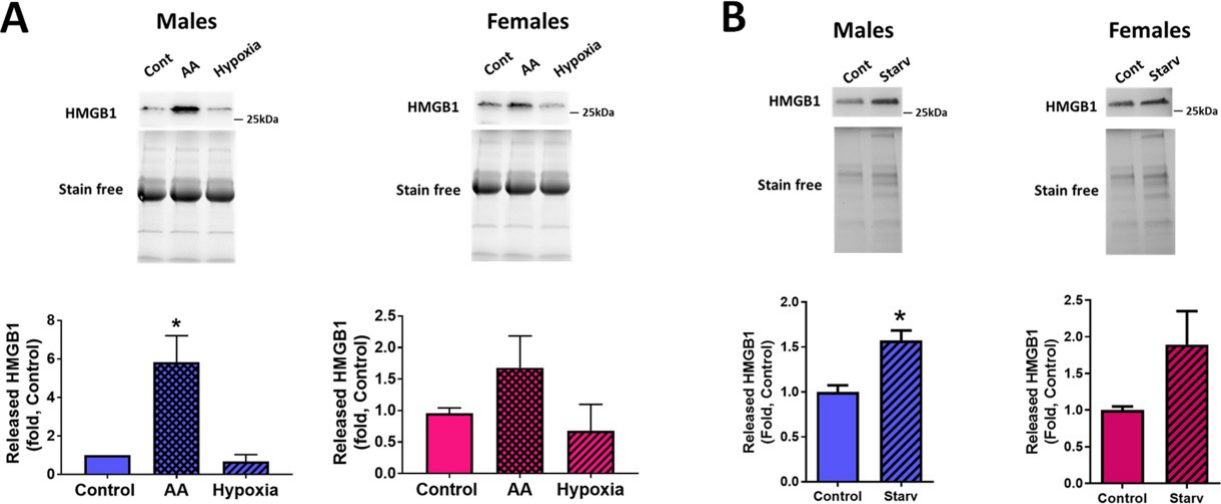
Sex difference in HMGB1 release in stressed MLEC and HPAEC. HMGB1 released into conditioned media was measured by WB analysis. **A**–HMGB1 signal in male and female MLEC exposed to hypoxia or AA as described above. **B**–HMGB1 in media collected from Control and starved male and female HPAEC. Values are means ± SEM. N = 4 repetitions for MLEC (5–7 mice isolated for each sex). *p < 0.05 versus Control MLEC. Statistical analysis was performed using one way ANOVA (Turkey’s multiple comparisons test). N = 6 repetitions for HPAEC. *p < 0.05 versus Control HPAEC. Statistical analysis was performed using the unpaired t-test.

Dying cells are known to release damage-associated molecular pattern molecules (DAMPs), such as high mobility group box 1 (HMGB1). Since apoptotic cells do not produce HMGB1 because it remains tightly attached to the apoptotic chromatin[26], necrotic or necroptotic cells are the primary source of HMGB1 that initiates a cascade of inflammatory responses through the binding to pattern recognition receptors and their activation [27]. Based on the discovered sex difference in the proportion of cells dying by necrosis and apoptosis, we investigated whether there is also a difference in the amount of HMGB1 released from male and female pulmonary endothelial cells. We confirmed that exposure of MLEC to hypoxia that was not associated with any necrosis did not produce any increase in the amount of released HMGB1 (Fig 7A). In contrast, AA treatment was associated with an increase in extracellular HMGB1, although male cells produced a significant, about a six-fold increase in HMGB1 levels, while females–only a slight insignificant elevation (Fig 7A). The same trend was found for HPAEC (Fig 7B).

## 4. Discussion

Multiple epidemiological registries confirmed the critical role of sex in PAH incidence and progression. Thus, the female prevalence is generally reported to be from 2:1 to 4:1 [28, 29]. At the same time, males have a worse survival prognosis [30, 31]. Our research team was the first to report that in the preclinical rat model, PAH manifests differently in males and females [14]. We proposed the presence of two gender-specific PAH phenotypes–a highly proliferative female phenotype that is associated with severe pulmonary artery remodeling; and a pro-inflammatory/ pro-fibrotic male phenotype. Indeed, male PAH rats develop severe perivascular inflammation in lungs and fibrotic changes in the vascular wall of small pulmonary arteries and right ventricle (RV) myocardium. These changes are absent in the female rats with PAH despite the similar increase in RV systolic pressure (RVSP) in both genders. The male sex is commonly associated with a more pronounced inflammatory response and a worse survival prognosis reported for different acute inflammatory diseases [7–10]. Thus, inflammatory changes in PAH seen precisely in males may be the result of the same predisposition of males to intensified inflammatory/immune system activation. However, the particular mechanisms that could be involved in male’s predisposition to the amplified inflammatory response remain unclear.

On most occasions, sex-mediated differences are attributed to the effects of sex hormones and their active metabolites [29, 32, 33], and both pathogenic and protective effects of hormones were reported to be directly implicated in PAH. However, the multiple controversies in the field suggest that other mechanisms could also be involved. Thus, the contribution of genomic factors was confirmed in the study showing that mice with XY chromosomes develop a more severe inflammatory response than an XX regardless of gender and independent of sex hormones [34]. Another recent study proposed the critical role of estrogen-independent activation of regulatory T cell (Treg) in females in their ability to control immune dysregulation and inflammation and protect endothelial cells [16].

Thus, not only hormonal stimulation but also other factors are involved in the interaction between the cells. For example, cytokines produced by T cells could mediate the sex-specific response and the outcome. It is unclear, though, whether this endocrine/paracrine stimulation fully defines the sex-different behavior of pulmonary vascular cells. To answer this question, we designed a study in which we studied the pulmonary endothelial cells omitted from the influence of sex hormones and various cell-cell interactions. We discovered that isolated male and female MLEC preserve the difference in their ability to respire, grow, and respond to cellular stresses even in the absence of humoral stimulation. According to our knowledge, this is the first time these aspects of sex differences of pulmonary endothelial cells were confirmed to be independent of either endocrine or paracrine factors.

Nevertheless, some of the previous research suggests that such a sex difference is possible. For example, in accordance with our finding that male MLEC and HPAEC show a higher proliferation rate, male smooth muscle cells [35], male neurons [36], and male liver cells [37] were found to proliferate quicker compared to females. The sex difference in neuron cell proliferation was blunted by stimulating female neurons with estradiol, which accelerated female cell growth to male levels [36]. This data suggests that the initial sexual dimorphism in the neuronal cell growth was due to genetic differences rather than sex hormones, which would only diminish a sex bias in cell proliferation rate. However, the degree of this effect *in vivo* could vary during the oestrus cycle.

It has been previously confirmed that the resting metabolic rate (RMR), which determines the amount of energy required to maintain normal homeostatic functions, is higher in men compared to women and independent of fitness level, menopausal status or age [38]. At the same time, females show a higher activity of mitochondrial oxidative phosphorylative complexes [39]. The more efficient mitochondrial machinery and bioenergetics in females are attributed to maternal transmission of mitochondria. Indeed, since mitochondrial DNA encodes only 13 out of 92 mitochondrial proteins involved in oxidative phosphorylation, efficient mitochondrial-nuclear communication is essential for mitochondrial function and energy production. In females, the evolutionary optimized mitochondrial/genetic regulation ensures more efficient mitochondrial function compared to males [40, 41]. Therefore, the upregulated level of mitochondrial respiration and ATP production in male MLEC and HPAEC discovered in our study may represent the attempt of the male cells to compensate the less efficient mitochondrial work and at the same time to maintain the adequate energy supply in cells with a higher level of energy demand. The disproportion seen in MLEC between 56% increase in ATP production and 119% increase in proton leak, a measure of uncoupling between substrate oxygen, and ATP generation in male cells additionally confirm the lower efficiency of male mitochondria. Interestingly, we did not see the same disproportion in HPAEC that instead show a comparable difference in ATP production in proton leak between males and females, which may relate to the better mitochondrial coupling in human cells compared to mice, although it may also be related to the particular profile of the human donors used, as discussed above.

The actively respiring mitochondria are known to be the primary source of reactive oxygen species (ROS) and the risk of cell damage. Therefore, we were interested in comparing the ability of the male and female cells to tolerate the different types of cellular stresses. Our results suggest that each sex has a predominant or ‘preferred’ cell death pathway. Thus, female pulmonary endothelial cells show a preference to die by apoptosis regardless of cell origin (mice or human) or type of the stress (hypoxia, inhibition of mitochondrial respiration by AA, or cell starvation). In contrast, male MLEC were found resistant to the hypoxic environment but responded to AA treatment or cell starvation with slightly increase apoptotic and severely enhanced necrotic cell death. Thus, it seems that male cells are more tolerable to the conditions associated with reduced ATP demand and reduced metabolism, like hypoxia [42]. At the same time, male cells are more sensitive to the situation when the ATP demand remains the same but can not be fulfilled, for example, due to the impairment in mitochondrial respiration (by AA) or inadequate supply of nutrients (cell deprivation). Indeed, it was noticed before that energy deprivation leads to caspase-independent necrotic cell death [43]. Importantly, depolarization of mitochondrial membranes is a critical event that precedes the rupture of the cytoplasmic membrane in dying cells [43], suggesting that the mitochondrial insufficiency plays a critical role in this process. Interestingly, antioxidants suppressed mitochondrial depolarization and subsequent necrosis. Since increased ROS production and reduced antioxidant protection is known to be strongly associated with male sex [44, 45], these factors could additionally predispose male cells to die by necrosis.

The discovery of sexual dimorphism in the cell death of isolated pulmonary endothelial cells strongly corresponds with a few other reports showing similar effects in other cell types. Thus, in male bone marrow-derived macrophages, the level of necrotic cell death in response to treatment with hydrogen peroxide was higher compared to females [46]. In primary cultured neurons, male cell death was also found to be caspase-independent, while female’s—caspase-dependent [47]. We have previously observed a sex bias in the amount of apoptotic and necrotic cells in the kidney of spontaneously hypertensive rats (SHR), with male SHR showing a greater necrotic cell death and female—more apoptotic [48]. However, in some of these studies, cells were analyzed right after their isolation from animals or used as tissue slices; thus, the potential effects of sex hormones and other paracrine factors could not be fully excluded. By confirming that these sex dimorphic effects stay preserved in cells isolated and cultured for a few passages we suggest the genetic factors rather than any extracellular stimuli are responsible for the sex-specific type of cell death. This conclusion is strongly supported by the evidence that this effect seems to be common for different cell types [46–48].

The difference in the type of activated cell death pathway has an important contribution in the signaling mediated by dying cells. The last, in turn, affects the survived neighboring cells. Thus, the release of damage-associated molecular patterns (DAMPs) from dying cells could induce a strong inflammatory response and immune system activation. High mobility group box 1 (HMGB1) is a DAMP that specifically releases from necrotic but not apoptotic cells [49]. Through its binding to pattern recognition receptors, such as Toll Like Receptor 4 (TLR4) and the receptor of advanced glycation end products (RAGE), HMGB1 activates the NfκB pathway and has been shown to play a critical role in the pathogenesis of PAH [27, 50]. In this study, we confirmed that the activation of necrotic cell death induced the release of HMGB1 in the culturing media. Importantly, the accumulation of HMGB1 protein was significant only in the media collected from male MLEC or HPAEC, while female cells showed a mild and non-significant response. This data is in a strong accordance with our translational study showing a significant increase in circulating HMGB1 only in male but not female PAH patients [15]. Taken together, our results suggest that the presence of non-hormonal factors is responsible for the sex difference in stress tolerance and stress response. This difference predisposes the male sex to over-activation of the inflammatory response and could contribute to the pathogenesis of PAH and other diseases.

The contribution of sex in cellular function and cellular fate is currently under a close investigation of many research groups. However, numerous contradictory conclusions suggest the involvement of not only hormonal regulation but also non-hormonal factors in the manifestation of sex disparity. The presence of several levels of regulation makes the system extremely complicated. To simplify this system, we evaluated the sex difference in the cells omitted from any extracellular regulation. We confirmed that sex difference in the proliferation rate, mitochondrial function, preferable type of cell death stress, and cell death signaling stay preserved in the isolated and cultured pulmonary endothelial cells. We propose that these processes could be under control of the cellular genomics, although additional studies are required to confirm this suggestion or identify the particular genes and downstream signaling pathways responsible for sex-specific regulation.

## Supporting information

S1 Raw images (PDF)
