# Supplementary material for: Sex-specific stress response and HMGB1 release in pulmonary endothelial cells
Source: PLoS One. 2020 Apr 9;15(4):e0231267. doi: 10.1371/journal.pone.0231267 (PMC7145198; doi:10.1371/journal.pone.0231267)

Figure 7A

MLEC isolated from male mice

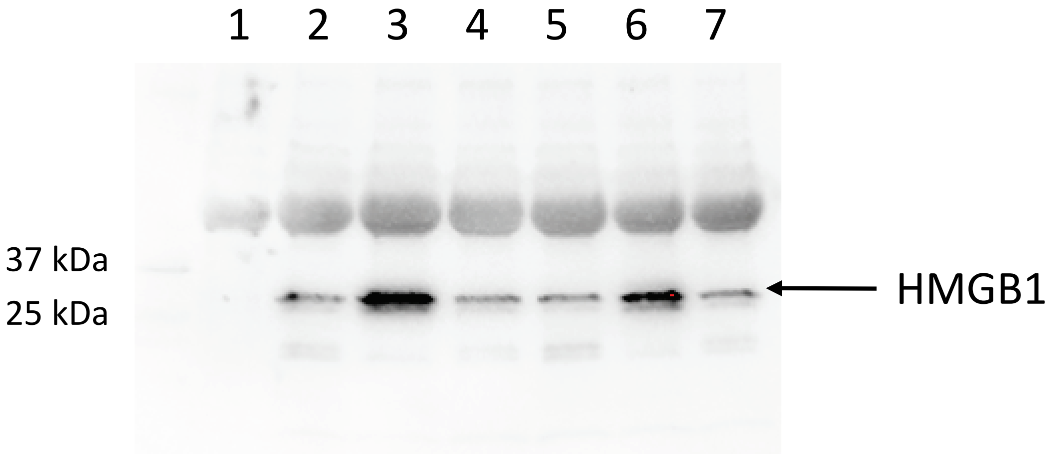

1 – Cell culture media never exposed to cells (negative Control)
2 - Control Male MLEC
3 – AA treated Male MLEC
4 – Hypoxia treated Male MLEC
5 – Control Male MLEC
6 - AA treated Male MLEC
7 - Hypoxia treated Male MLEC

Figure 7A

Stain Free image used for normalization of the protein loading
(MLEC from male mice)

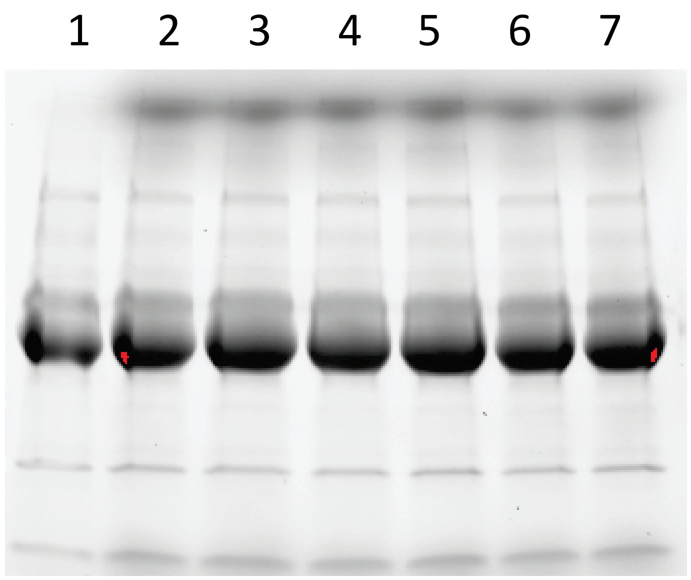

1 – Cell culture media never exposed to cells (negative Control)
2 - Control Male MLEC
3 – AA treated Male MLEC
4 – Hypoxia treated Male MLEC
5 – Control Male MLEC
6 - AA treated Male MLEC
7 - AA treated Male MLEC

Figure 7A

MLEC isolated from female mice

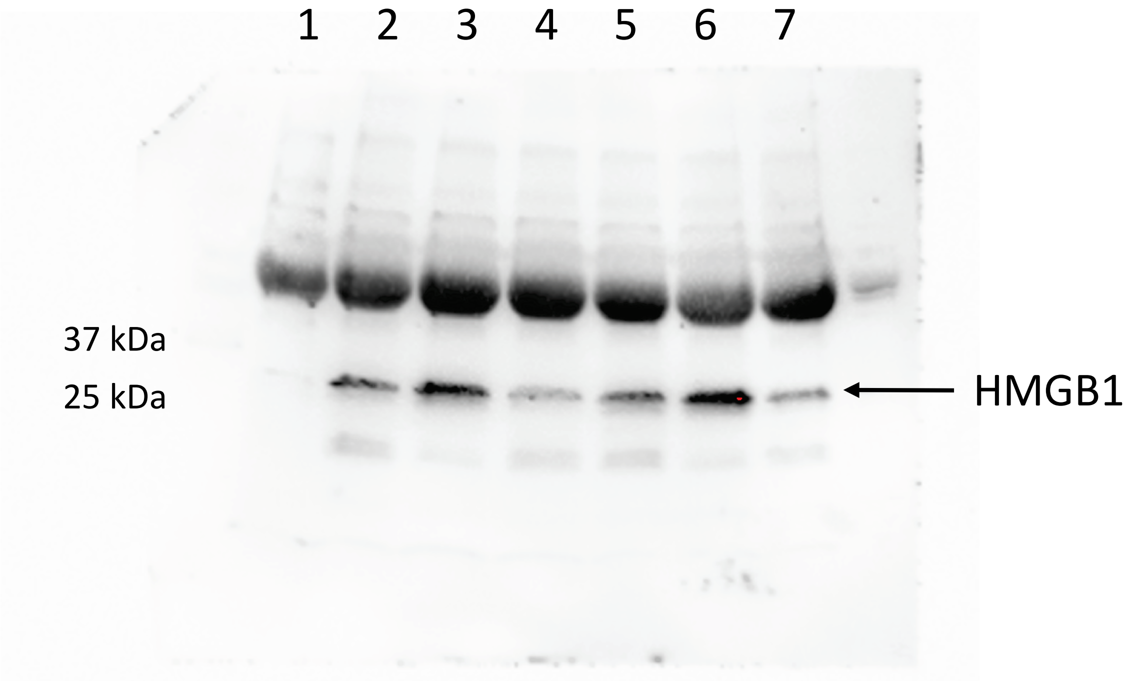

1 – Cell culture media never exposed to cells (negative Control)
2 - Control Female MLEC
3 – AA treated Female MLEC
4 – Hypoxia treated Female MLEC
5 – Control Female MLEC
6 - AA treated Female MLEC
7 - Hypoxia treated Female MLEC

Figure 7A

Stain Free image used for normalization of the protein loading
(MLEC from female mice)

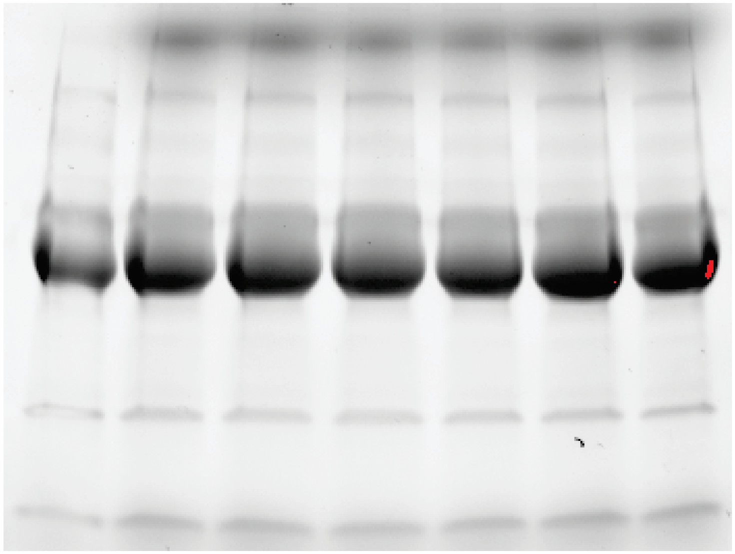

1 – Cell culture media never exposed to cells (negative Control)
2 - Control Female MLEC
3 – AA treated Female MLEC
4 – Hypoxia treated Female MLEC
5 – Control Female MLEC
6 - AA treated Female MLEC
7 - Hypoxia treated Female MLEC

Figure 7 B

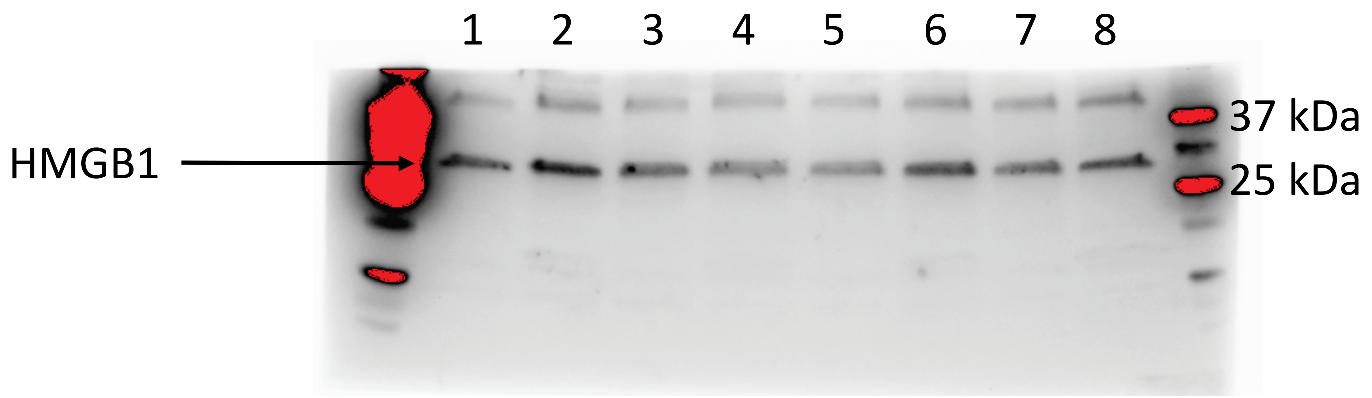

1 – Control Male HPAEC
2 – Starved Male HPAEC
3 – Control Female HPAEC
4 – Starved Female HPAEC
5 - Control Male HPAEC
6 - Starved Male HPAEC
7- Control Female HPAEC
8 - Starved Female HPAEC

Figure 7 B

Stain Free image used for normalization of the protein loading

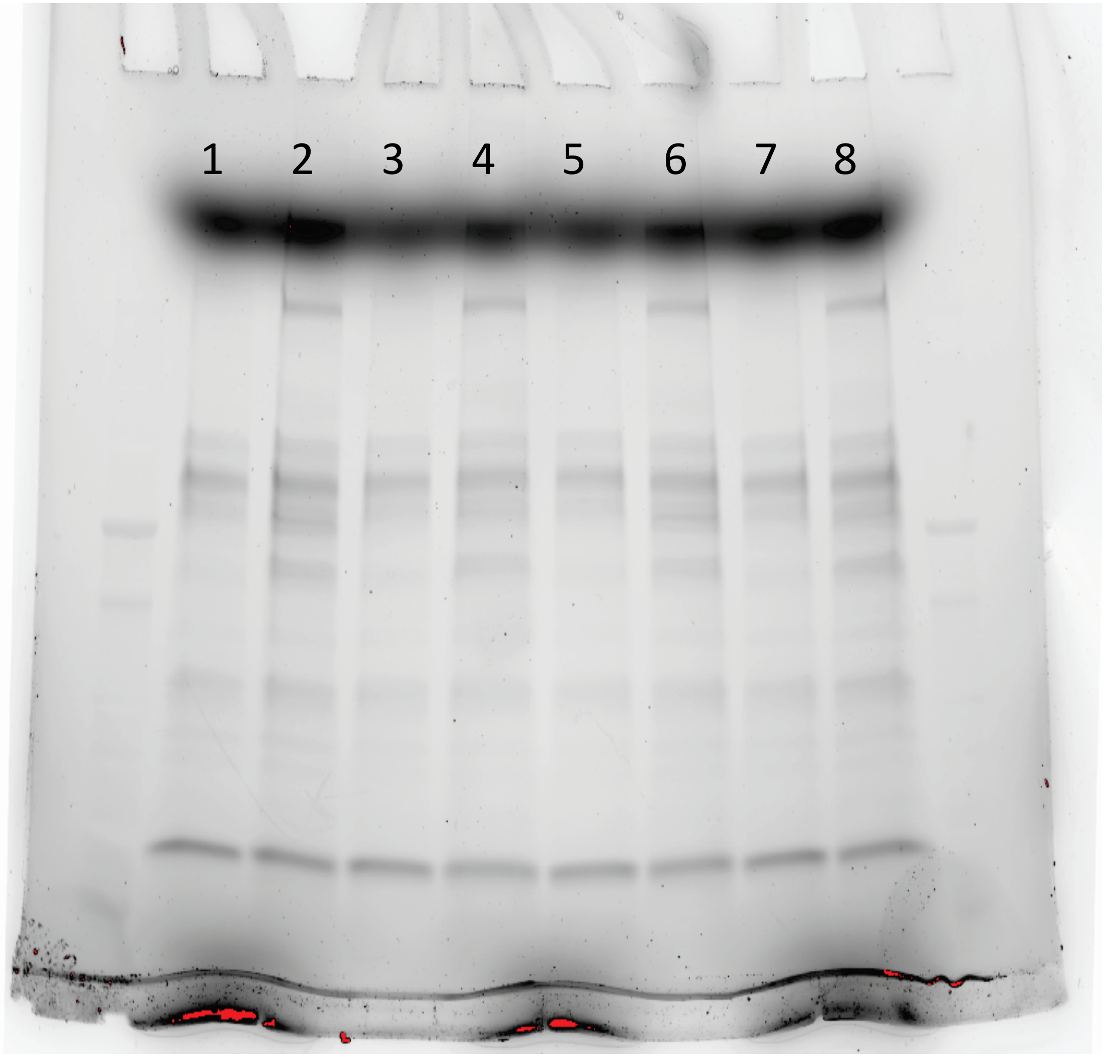

1 – Control Male HPAEC
2 – Starved Male HPAEC
3 – Control Female HPAEC
4 – Starved Female HPAEC
5 - Control Male HPAEC
6 - Starved Male HPAEC
7- Control Female HPAEC
8 - Starved Female HPAEC

Male MLEC stained by JC1

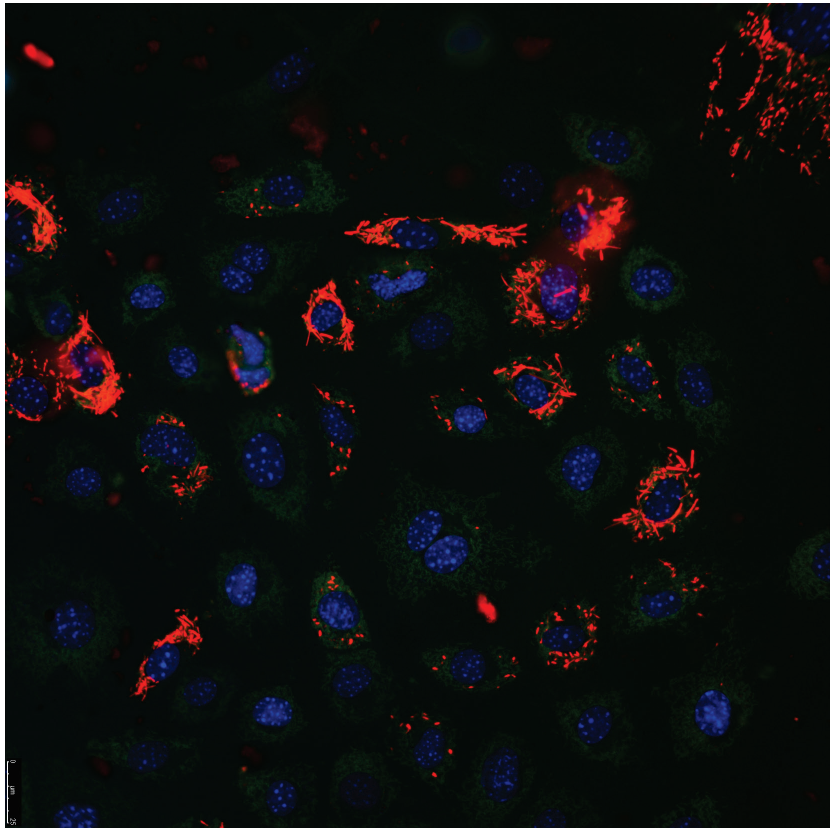

Female MLEC stained by JC1

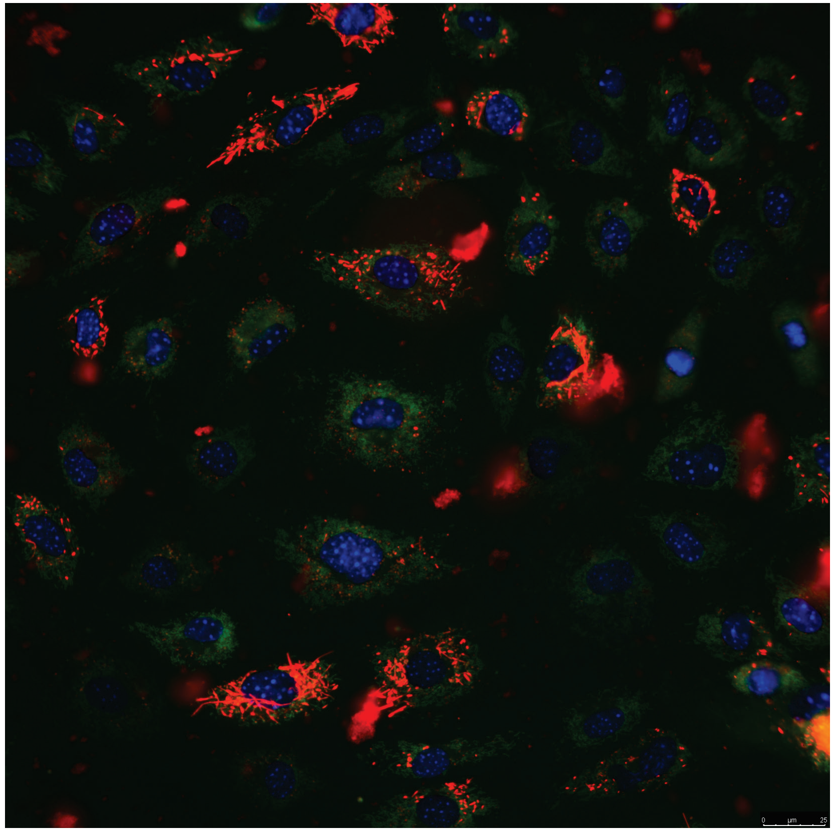

Supplement: S1 Raw images — (PDF) [file pone.0231267.s001.pdf]
